# Direct comparison of metabolic health effects of the flavonoids quercetin, hesperetin, epicatechin, apigenin and anthocyanins in high-fat-diet-fed mice

**DOI:** 10.1007/s12263-015-0469-z

**Published:** 2015-05-29

**Authors:** Elise F. Hoek-van den Hil, Evert M. van Schothorst, Inge van der Stelt, Hans J. M. Swarts, Marjanne van Vliet, Tom Amolo, Jacques J. M. Vervoort, Dini Venema, Peter C. H. Hollman, Ivonne M. C. M. Rietjens, Jaap Keijer

**Affiliations:** Human and Animal Physiology, Wageningen University, Wageningen, The Netherlands; Division of Toxicology, Wageningen University, Wageningen, The Netherlands; RIKILT Wageningen UR, Wageningen, The Netherlands; Laboratory of Biochemistry, Wageningen University, Wageningen, The Netherlands

**Keywords:** Bioactive food components, Polyphenols, Quercetin, Whole-body homeostasis, Lipid metabolism, Gene expression

## Abstract

Dietary flavonoid intake is associated with reduced risk of cardiovascular diseases, possibly by affecting metabolic health. The relative potency of different flavonoids in causing beneficial effects on energy and lipid metabolism has not been investigated. Effects of quercetin, hesperetin, epicatechin, apigenin and anthocyanins in mice fed a high-fat diet (HF) for 12 weeks were compared, relative to normal-fat diet. HF-induced body weight gain was significantly lowered by all flavonoids (17–29 %), but most by quercetin. Quercetin significantly lowered HF-induced hepatic lipid accumulation (71 %). Mesenteric adipose tissue weight and serum leptin levels were significantly lowered by quercetin, hesperetin and anthocyanins. Adipocyte cell size and adipose tissue inflammation were not affected. The effect on body weight and composition could not be explained by individual significant effects on energy intake, energy expenditure or activity. Lipid metabolism was not changed as measured by indirect calorimetry or expression of known lipid metabolic genes in liver and white adipose tissue. Hepatic expression of *Cyp2b9* was strongly downregulated by all flavonoids. In conclusion, all flavonoids lowered parameters of HF-induced adiposity, with quercetin being most effective.

**Electronic supplementary material:**

The online version of this article (doi:10.1007/s12263-015-0469-z) contains supplementary material, which is available to authorized users.

## Introduction

Fruit and vegetable consumption is regarded as protective against cardiovascular diseases (CVD) (Estruch et al. 2013). Flavonoids in fruits and vegetables are suggested to be responsible, at least partly, for these protective effects. This is supported by epidemiological studies that have shown that an increased intake of flavonoids is associated with a reduction in CVD risk (Peterson et al. 2012). Nowadays flavonoid supplements are widely available, resulting in even increasing intakes of flavonoids. Elevated circulating lipid levels, a fatty liver and obesity are associated with a higher risk of CVD (Boden 2008; Harchaoui et al. 2009; Scorletti et al. 2011). Previously, we have shown that the flavonoid quercetin can lower serum lipid levels by affecting hepatic lipid metabolism (Hoek-van den Hil et al. 2013) and that quercetin reduced high-fat-diet-induced body weight gain and hepatic lipid accumulation (Hoek-van den Hil et al. 2014).

Quercetin is one of the many flavonoids in our diet and is the major representative of the flavonoid subclass of flavonols. Other flavonoid subclasses are flavanones, flavan-3-ols, flavones, isoflavones and anthocyanins, which all are present in our diet. However, it is not known whether these flavonoids exert similar effects as quercetin on body weight homeostasis and lipid metabolism, and if so, whether they affect the same metabolic pathways. Human intervention studies with fruits or vegetables or extracts rich in flavonols, flavanones, flavan-3-ols or anthocyanins showed beneficial effects on CVD-related parameters such as blood pressure, vascular function or blood lipid profiles (Chong et al. 2010; Hooper et al. 2008; Massaro et al. 2010). Also, flavones, mostly present in herbs and cereals, are suggested to have beneficial effects on CVD risk factors (Benavente-Garcia and Castillo 2008). Therefore, it is interesting to compare the effects of flavonoids from different subclasses on CVD risk factors.

The observed potential beneficial effects of flavonoids in humans are mostly based on studies using flavonoid-rich foods or extracts (Kim et al. 2011; Perez-Vizcaino and Duarte 2010). Therefore, also other components can be responsible for the observed effects. For instance, caffeine was presumably present in studies with green tea catechin extracts, which could be underlying the reduction in body weight gain (Phung et al. 2010). Functional effects of flavonoids should therefore be investigated with fully characterised pure compounds. Furthermore, we have shown that effects of quercetin on body weight depended on the percentage and/or composition of fat in the background diet (Hoek-van den Hil et al. 2014). It is therefore important to directly compare effects using the same diet. A high-fat diet that induces body weight gain and hepatic lipid accumulation seems best suited to reveal the lipid lowering properties of flavonoids.

Flavonoids were selected from five subclasses, based on potential CVD protective effects, and presence in food (Benavente-Garcia and Castillo 2008; Chong et al. 2010; Hooper et al. 2008; Manach et al. 2005; Massaro et al. 2010). In addition to quercetin (flavonol), hesperetin (flavanone), epicatechin (flavan-3-ol), apigenin (flavone) and a purified extract of mainly cyanindin-3-O-β-glucoside and delphinidin-3-O-β-glucoside (anthocyanins) were selected. The aim of this study was to investigate whether other flavonoids exert similar effects as quercetin, which reduced high-fat-induced body weight gain and hepatic lipid accumulation (Hoek-van den Hil et al. 2014). To better understand the effects, we extend the assessment with analysis of whole-body energy balance and other metabolic health-related analyses. A comprehensive analysis of whole-body energy balance was performed, including quantification of parameters such as body weight gain, faecal energy loss, energy intake and activity. Indirect calorimetry was used to evaluate the type and rate of substrate utilisation and energy expenditure (EE). Furthermore, circulating lipids, hepatic lipid accumulation and hepatic gene expression patterns were studied. Additionally, the effects of the flavonoids on gene expression in white adipose tissue (WAT) were assessed, together with WAT-secreted serum leptin levels. Leptin is a peptide hormone which regulates body weight gain.

## Methods

### Animals and treatments

Eighty-four male C57BL/6JOlaHsd mice (Harlan Laboratories, Horst, The Netherlands) were individually housed under controlled conditions (temperature 21 °C, 12 h/12 h light–dark cycle, 55 ± 15 % humidity), with ad libitum access to food and water. At arrival, the mice were 9 weeks of age. During the first 5 days of a 3-week adaptation period, mice were fed a standard Harlan chow diet, followed by a standardised semi-synthetic normal-fat diet [NF, 10 energy% (en%) fat] with the same dietary constituents as the intervention high-fat diet (HF, 40 en%) in which carbohydrates were substituted with fats (Hoevenaars et al. 2012) (Research Diets Services B.V., Wijk bij Duurstede, The Netherlands). At the start of the 12-week intervention period, mice were stratified based on body weight over 7 groups (*n* = 12), to obtain identical groups for this important parameter. Male mice were used in order to enable comparison of outcomes with previous findings (Hoek-van den Hil et al. 2014). One group of mice continued on NF, while the other six groups of mice received HF with or without supplementation of different flavonoids (HF + flavonoids). A subset of data of the control HF and HF + quercetin group were published before (body weight, energy intake, serum quercetin levels, and serum and hepatic lipid levels) (Hoek-van den Hil et al. 2014). Flavonoids were added in equimolar amounts to HF (0.01 mol/kg diet), amounts were based on our previous results, which showed effectiveness of quercetin at this concentration (de Boer et al. 2006; Hoek-van den Hil et al. 2013): 0.33 % (w/w) quercetin (Sigma, Zwijndrecht, The Netherlands), 0.33 % hesperetin (Bioconnect, Huissen, The Netherlands), 0.32 % epicatechin (Sigma), 0.29 % apigenin (Fuzhou Corona Science & Technology Development Co., Ltd., Fuzhou Fujian, China) and 0.5 % anthocyanins, a purified anthocyanin extract from bilberry and blackcurrant consisting of mainly cyanindin-3-O-β-glucoside and delphinidin-3-O-β-glucoside (kindly provided by Medox, Polyphenols Laboratories, Sandnes, Norway; the exact composition of this extract has been described by Qin et al. 2009). Sufficient amounts of individual anthocyanins were not available. Body weight and food intake were monitored weekly. Faeces were collected in weeks 11 and 12. One HF + quercetin (HF + Q)-fed mouse was excluded from all analyses, because of a nasal abscess. At the end of the intervention, all mice were fasted for 2–4 h during the light phase and anesthetised by inhalation of 5 % isoflurane using O_2_ as a carrier. Blood was sampled via orbital extraction in collect serum tubes (Greiner Bio-one, Longwood, USA) and stored at −80 °C after obtaining serum. After blood collection, mice were killed by cervical dislocation, and liver, epididymal and mesenteric white adipose tissues (epiWAT and mesWAT, resp.) were dissected, weighted and snap frozen in liquid nitrogen and stored at −80 °C. The experiment was performed according to the Dutch Animal Experimentation Act (1996), and the experimental protocol was approved by the Animal Welfare Committee of Wageningen University, Wageningen, The Netherlands (DEC 2011079).

### HPLC analysis of flavonoid levels in serum and diet

Flavonoid levels in serum were measured using HPLC with coulometric array detection as described (Hoek-van den Hil et al. 2013). Anthocyanins could not be detected by our method. Before analysis, samples were hydrolysed by β-glucuronidase/sulphatase to obtain deconjugated flavonoids, resulting in total flavonoid levels being the sum of all glucuronidated and sulphated conjugates, while the methylated conjugates were separately quantified. Flavonoid levels in the diets were also measured with HPLC to confirm presence and stability.

### Energy content of faeces and diet

Bomb calorimetry was used to determine energy content of diet and faeces (Calorimeter C7000, IKA, Staufen, Germany) as described (Hoek-van den Hil et al. 2014). Total digestible energy intake over 12 weeks was calculated based on weekly energy intake and faecal energy loss, by multiplying weekly food intake by the measured dietary gross energy content minus the extrapolated faecal energy loss. Digestible energy intake was assumed to be comparable with metabolisable energy intake, as dietary protein content was equal for all diets and no differences in urinary energy losses were expected.

### Indirect calorimetric and activity measurements

Indirect calorimetry and activity were measured in weeks 1, 5 and 11. Indirect calorimetry was performed by an open-circuit LabMaster Metabolism Research Platform (TSE systems GmbH, Bad Homburg, Germany) and analysed as described previously (Hoevenaars et al. 2013) with minor adaptations. A reference cage was measured, and then, rates of oxygen consumption (VO_2_) and carbon dioxide production (VCO_2_) were measured during 1 min every 12 min for 48 h, of which the last 24 h were used. To avoid any influence of initial stress and adaptation, only the data of the last 24 h of a 48-h measurement were used for analysis. Respiratory exchange ratio (RER) is defined as VCO_2_ divided by VO_2_, and EE was calculated using the equation [3.815 + (1.232 × RER)] × VO_2_ (Mclean and Tobin 1987). Carbohydrate and lipid oxidation rates were calculated using (Peronnet and Massicotte 1991). During indirect calorimetry measurements, activity was continuously measured with the ActiMot system (TSE systems GmbH) in eight cages. Infrared beam breaks in horizontal plane (*x* and* y* direction) over the last 24 h of a 48-h period were used.

### Motor performance

Balance and motor coordination was assessed by Rotarod (IITC Life Science, Woodland Hills, USA) in week 9. Latency to fall was recorded on an accelerating rod (3–38 rpm in 300 s); mice were placed on the rod four times with an inter trial rest period of 30 min; and the average of two longest runs per animal were used for analysis.

Several parameters of gait were assessed in week 10 by CatWalk analysis (Noldus Information Technology, Wageningen, The Netherlands) using the reflection of light projected on a glass walking area. Each mouse made at least six compliant runs, being defined as a maximum speed variation of 40 %, minimum run duration of 0.5 s and maximum run duration of 10 s. Quantitative gait parameters were analysed using the CatWalk XT 10.0 software (Noldus Information Technology).

### Lipid determination in serum and liver

Because flavonoids were previously shown to interfere with commonly used commercially available enzymatic lipid assays (Hoek-van den Hil et al. 2012), alternative methods were used to measure the amount of lipids in serum and liver, as described (Hoek-van den Hil et al. 2013). Serum lipids were extracted and analysed with ^1^H nuclear magnetic resonance (^1^H-NMR), and neutral lipids were stained in frozen liver sections with Oil red O (Sigma) and quantified.

### RT-qPCR

RNA from liver was isolated using RNeasy columns (Qiagen, Venlo, The Netherlands), RNA from epiWAT was extracted with Trizol (Invitrogen, Breda, The Netherlands), and quality was verified [as published (Hoek-van den Hil et al. 2013; Hoek-van den Hil et al. 2014)]. RT-qPCR was performed and analysed as described [6]. Data were normalised using reference genes beta-2 microglobulin (*B2m*) and hypoxanthine phophoribosyltransferase 1 (*Hprt1*) for liver and *Hprt1* and Ribosomal protein S15 (*Rps15*) for epiWAT, chosen based on stable gene expression levels as determined with GeNorm (GeNorm, Ghent University Hospital, Ghent, Belgium). Primer sequences can be found in Supplementary Table S1.

### Serum leptin levels

Serum leptin levels were determined using a leptin ELISA kit (Crystal Chem Inc., Chicago, IL, USA) according to the manufacturer’s instructions.

### Histology of epididymal white adipose tissue

Paraffin-embedded epididymal white adipose tissue (epiWAT) was cut into 5-µm sections and stained using Periodic Acid Schiff Haematoxylin (PASH). Per animal, circumference of at least 400 adipocytes was measured using AxioVision software v4.8 (Carl Zeiss Microscopy GmbH, Jena, Germany). To estimate macrophage infiltration as a marker for tissue inflammation, a MAC-2 staining was performed and analysed as published (Hoevenaars et al. 2014). Macrophage infiltration is expressed as total number of crown-like structures (CLS) per 100 adipocytes; CLS are formed by macrophages around dying or dead adipocytes.

### Statistical analysis

All statistical analyses were in principle done based on all 12 mice per group with the following exceptions: HF + Q only for 11 mice; for indirect calorimetry measurements (*n* = 9), histological stainings (*n* = 6), faeces collection (*n* = 4) and serum flavonoid measurements (*n* = 6), subsets of mice as indicated between the brackets were randomly selected, because of limited equipment or practical reasons. GraphPad Prism version 5.03 (GraphPad software, San Diego, CA, USA) was used for statistical analysis. Data were checked for normality and if needed log transformed (*Acot3*, *Cyp4a14*, *Por*, *Fasn* and *Cyp2b9* in liver and *Cpt1a* in EpiWAT). One-way ANOVA was used to compare the groups, followed by Dunnett’s post hoc test to compare the different HF + flavonoid groups to HF, and HF to NF. For the analysis of data of liver weight, hepatic gene expression of *Acacb* and *Cpt1a*, and WAT gene expression of *Pparg,* a Kruskal–Wallis test was used, because also after log transformation these data were not normally distributed. Curve fitting was used to analyse body weight gain during the 12-week intervention period using PROAST software (Slob 2002). Two-way ANOVA (no repeated measures) was used for analysis of the lipid profiles in serum and for 24-h indirect calorimetry data in time. Pearson correlation analyses were performed using all HF + flavonoids groups and HF group. NF was excluded for these correlation analyses, to avoid false-positive strong correlations due to NF. *p* values smaller than 0.05 were considered significantly different.

## Results

### Flavonoid quantification in diets and serum

Flavonoids in the diets were measured at the start of the experiment and were between 87 and 99 % of the theoretical amounts. After 1 week at room temperature in the cages, contents of flavonoids in the diets did not change and varied between 90 and 100 % of the theoretical flavonoid amounts (Supplementary Table S2).

After 12 weeks on the HF + quercetin diet (HF + Q), the sum of quercetin and isorhamnetin in serum was 6.5 ± 1.4 µM (Hoek-van den Hil et al. 2014). After the HF + hesperetin diet (HF + H), hesperetin was detectable in half of the measured serum samples (with a detection limit of 150 nM), for which the mean concentration was 0.5 ± 0.8 µM. Epicatechin and apigenin were not detectable in serum of HF + epicatechin (HF + E) and HF + apigenin (HF + Ap) mice (with detection limits of 300 nM).

### Body weight, metabolisable energy intake and feed efficiency

**Fig. 1:**
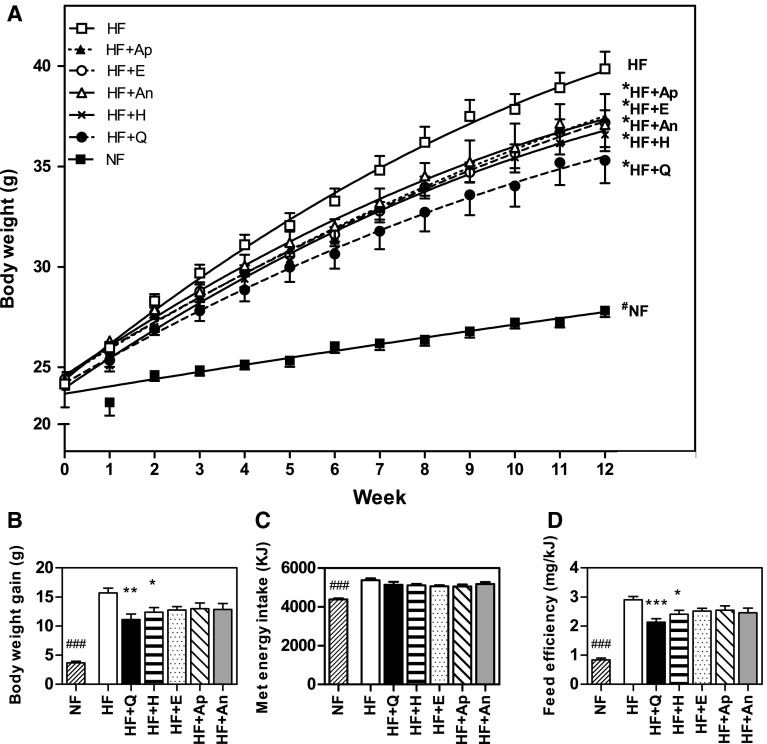
Flavonoids reduce HF-induced body weight gain and feed efficiency. Body weight of the mice during 12 weeks on a normal-fat diet (NF, 10 en% fat) or a high-fat diet (HF, 40 %) with or without supplementation of the flavonoids quercetin (HF + Q), hesperetin (HF + H), epicatechin (HF + E), apigenin (HF + Ap) or anthocyanins (HF + An) (**a**). Total body weight gain over 12 weeks (**b**). Total metabolisable energy intake over 12 weeks (**c**). Total food efficiency, which is the ratio of body weight gain over metabolisable energy intake (**d**). Data are presented as mean ± SEM. *Asterisk* indicates a significant difference of HF + flavonoid to HF (*p* < 0.05), **p < 0.01, ***p < 0.001, *hash* indicates a significant difference of HF to NF (*p* < 0.05), ^###^ *p* < 0.001

Body weight was significantly increased due to HF compared with NF feeding during the whole intervention period, with a cumulative body weight gain of HF mice being four times higher than the weight gain of NF mice (Fig. 1). Supplementation of HF with any of the flavonoids (HF + flavonoids) reduced the body weight gain significantly, as analysed by curve fitting analysis (Fig. 1a). Cumulative body weight gain over 12 weeks was for HF + Q mice 29 % lower (*p* < 0.001) (Hoek-van den Hil et al. 2014) and for HF + H mice 21 % lower (*p* < 0.05) than for HF mice (Fig. 1b). The cumulative metabolisable energy intake over 12 weeks was not significantly different for all HF + flavonoid groups compared with HF, but it was significant different for HF versus NF (Fig. 1c). As a result, feed efficiency was significant lower for HF + Q, HF + H and NF compared with HF (Fig. 1d). The calculated flavonoid intake based on the food intake of the flavonoid-fed mice was for quercetin ~350 mg/kg bw/day, for hesperetin ~320 mg/kg bw/day, for epicatechin ~300 mg/kg bw/day, for apigenin ~500 mg/kg bw/day and for anthocyanins ~500 mg/kg bw/day.

### Indirect calorimetry and activity

**Fig. 2:**
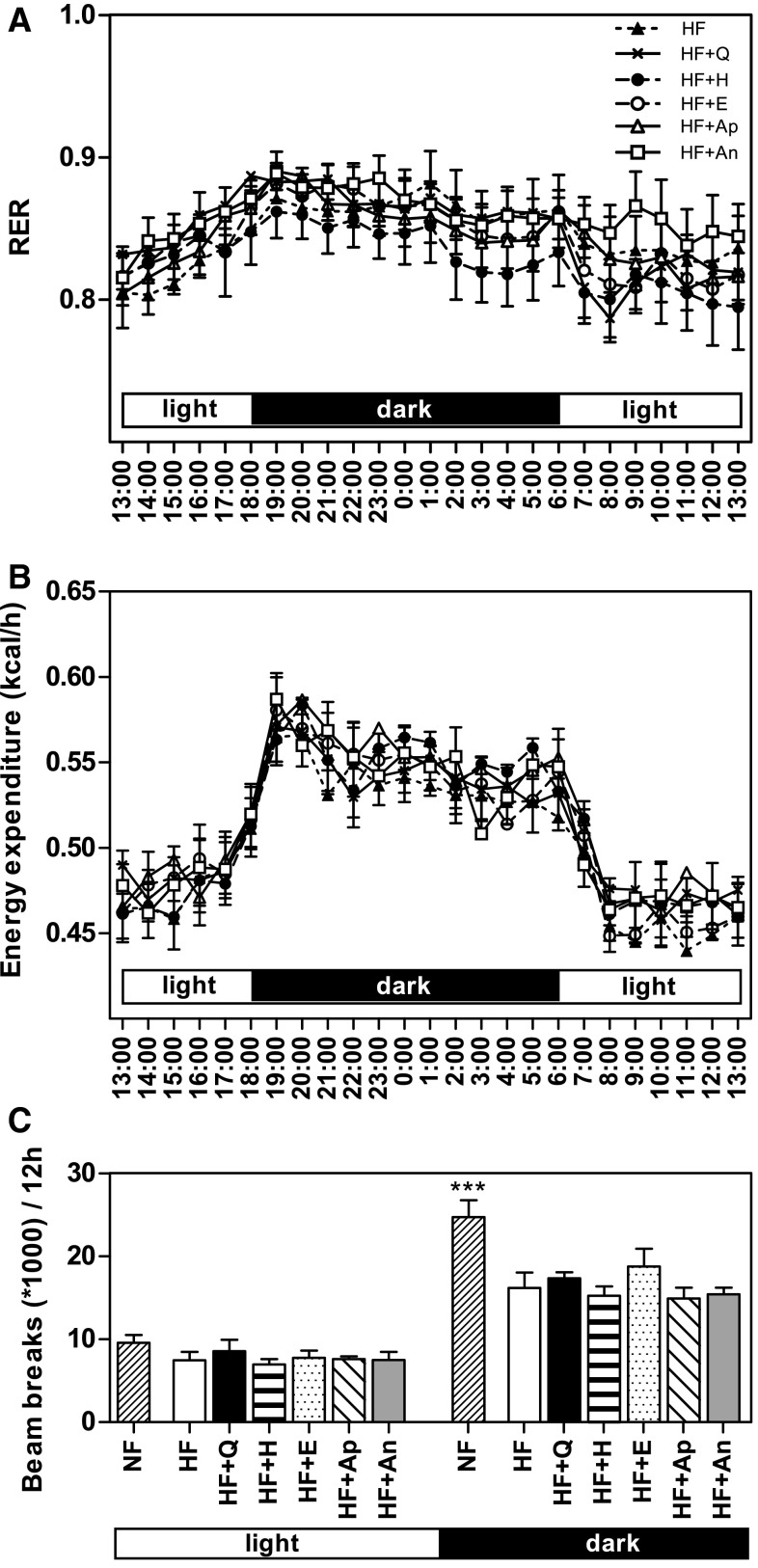
Whole-body RER, EE and activity levels were not affected by flavonoid supplementation. Indirect calorimetry measurements of 24-h RER (**a**) and EE (**b**) in week 11. Activity measured during indirect calorimetry measurements for light (12-h) and dark (12-h) period (**c**). Data are presented as mean ± SEM. *Double hash* indicates a significant difference of HF to NF (*p* < 0.001). RER, respiratory exchange ratio; EE, energy expenditure; NF, normal-fat diet; HF, high-fat diet; HF + Q, HF supplemented with quercetin; HF + H, hesperetin; HF + E, epicatechin; HF + Ap, apigenin; HF + An, anthocyanins

Respiratory exchange ratio (RER) and EE were not significantly different between any HF + flavonoid group and HF in week 11, the 24-h patterns are shown in Fig. 2a, b. Mean 12-h light- and dark-phase RER and EE values are in Supplementary Table S3. Mean RER values varied from 0.84 to 0.87, which implies that 48.3–58.5 % of the energy comes from glucose oxidation and 51.7–41.5 % from fat oxidation. HF mice had a significant lower RER compared with NF, which is caused by the lower carbohydrate and higher fat levels present in HF. EE was not significant different between HF and NF mice. HF mice were significantly less active during the dark phase compared with NF mice. No differences were observed for activity of HF + flavonoids groups compared with HF during the dark phase as well as the light phase (Fig. 2c). RER, EE and activity for weeks 1 and 5 were all comparable to those of week 11 (Supplementary Figure S1).

### Motor performance

HF mice performed significantly poorer on the Rotarod than NF mice, likely due to their higher body weight. Performance on Rotarod was, however, not significantly different between any HF + flavonoid group and HF (Supplementary Figure S2). There were also no significant differences between the HF + flavonoid groups and HF in the measured gait parameters (Supplementary Table S4); however, some parameters showed significant differences between HF and NF, also likely related to the large body weight differences.

### Serum lipids

**Fig. 3:**
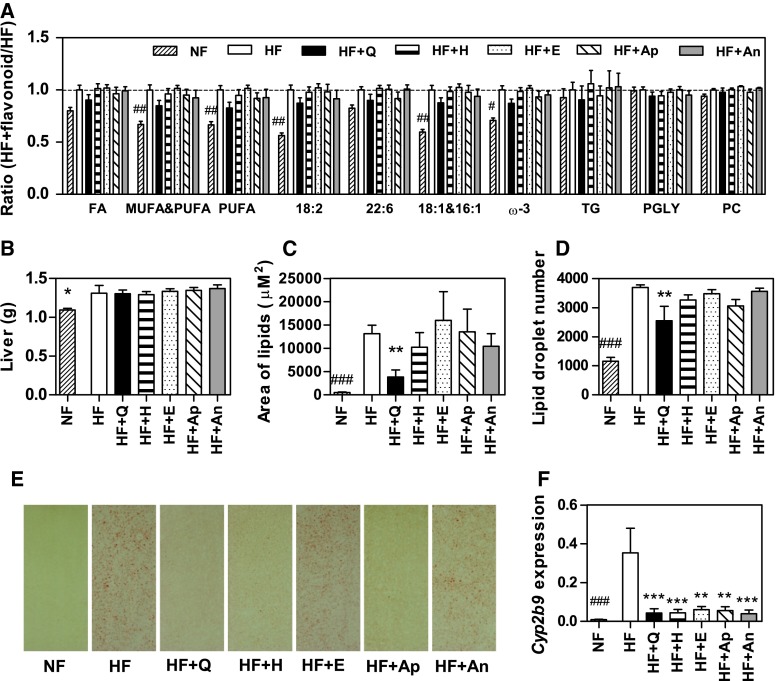
Effects of flavonoids on HF-induced effects on serum lipids, liver weight and hepatic lipid accumulation. Serum lipid fractions are shown as ratio of HF + flavonoid or NF over the average value of the HF control (**a**). Liver weight at the end of the dietary intervention of 12 weeks (**b**). Quantification of total area of lipids per picture (**c**) and mean lipid droplet number per picture (**d**) in liver. Representative pictures of stained hepatic lipids by Oil red O (**e**). Hepatic Cyp2b9 expression (**f**). Data are presented as mean ± SEM. *Asterisk* indicates a significant difference of HF + flavonoid to HF; *hash* indicates a significant difference of HF to NF (*p* < 0.05); ^##^ *p* < 0.01. PUFA, poly unsaturated fatty acids; MUFA, mono unsaturated fatty acids; FA, fatty acids; TG, triglycerides; PGLY, phosphoglycerides; PC, phosphatidylcholine; EC, esterified cholesterol; TC, total cholesterol; NF, normal-fat diet; HF, high-fat diet; HF + Q, HF supplemented with quercetin HF + Q; hesperetin, HF + H; epicatechin, HF + E; apigenin, HF + Ap; anthocyanins, HF + An

HF significantly induced higher levels of ‘MUFA and PUFA’, ‘PUFA’, ‘18:2 fatty acids’, ‘18:1 and 16:1 fatty acids’ and ‘omega 3 fatty acids’ in serum compared with NF. No significant differences between HF and HF + flavonoid were observed in the serum lipid levels; however, some of the flavonoids showed a small trend towards the levels found for NF (Fig. 3a).

### Hepatic lipid accumulation

HF feeding significantly induced hepatic lipid accumulation as is apparent from an increase in liver weight (Fig. 3b), area of lipids (Fig. 3c) and lipid droplet number (Fig. 3d) compared with values for NF feeding. Liver weight was not affected by the flavonoid supplementations to the diets. Only supplementation with quercetin (HF + Q) significantly reduced the HF-induced lipid accumulation. As compared to HF, quercetin lowered the area of lipids by 71 %, and the lipid droplet number by 31 %. Representative pictures of hepatic lipid stainings are presented in Fig. 3e.

### Hepatic gene expression

Hepatic expression levels of genes involved in fatty acid omega-oxidation were studied based on our previous observations that quercetin supplementation to a mild high-fat (30 en%) diet induced this pathway in liver (Hoek-van den Hil et al. 2013). HF feeding compared with NF revealed significant induction of omega-oxidation by regulation of *Acot3*, *Cyp4a10* and *Cyp4a14.* However, no significant regulation of these genes, nor *Por* was observed in any of the HF + flavonoid groups compared with HF (Table 1). Subsequently, also other genes involved in lipid metabolism were studied. *Acacb* and *Ppargc1a* were significantly regulated by HF versus NF. However, *Acacb*, *Fasn*, *Cpt1a*, *Ppara* and *Ppargc1a* were not significantly different for any of the HF + flavonoid diets compared with HF (Table 1).

**Table 1:** Hepatic gene expression (RT-qPCR)

Gene symbol	HF	HF + Q	HF + H	HF + E	HF + Ap	HF + An
	versus NF	versus HF				
Lipid omega-oxidation						
*Acot3*	2.67^##^	1.07	−1.07	1.07	1.16	1.05
*Cyp4a10*	2.06^##^	−1.09	1.15	1.05	−1.12	1.05
*Cyp4a14*	2.39^###^	1.11	1.23	1.09	1.06	1.04
*Por*	1.34	1.08	1.14	1.12	1.07	1.02
Lipid metabolism						
*Acacb*	−1.76^##^	1.07	1.01	1.11	−1.10	1.12
*Fasn*	−1.10	1.10	1.05	1.17	−1.05	1.02
*Cpt1a*	1.25	−1.06	1.05	−1.02	−1.12	−1.01
*Ppara*	1.29	−1.07	1.09	1.02	−1.09	1.01
*Ppargc1a*	−1.51^##^	−1.06	1.05	1.10	1.18	−1.09
CAR target						
*Cyp2b9*	40.60^###^	−8.22***	−8.02***	−5.85**	−6.33**	−9.05***

Data are presented as mean fold change of HF versus NF and HF + flavonoids versus HF

NF, normal-fat diet; HF, high-fat diet; HF supplemented with quercetin, HF + Q; hesperetin, HF + H; epicatechin, HF + E; apigenin, HF + Ap; anthocyanins, HF + An

^##^Indicates significant difference of HF to NF (*p* < 0.01), ^###^ *p* < 0.001, ** indicates a significant difference of HF + flavonoid to HF (*p* < 0.01), *** *p* < 0.001

*Cyp2b9*, a target gene of the transcription factor Constitutive Androgen Receptor (CAR also known as NR1I3), was strongly upregulated by HF versus NF. Interestingly, this gene was significantly lower expressed upon supplementation of HF with all of the flavonoids (FC between −5.9 and −9.1, Table 1). *Cyp2b9* expression levels for all groups are shown in Fig. 3f.

### Gene expression in white adipose tissue

Based on the above-described results, it was decided to study gene expression in epiWAT only for NF, HF, HF + Q and HF + H groups. Genes were selected by their functions related to lipid metabolism. This showed significant regulation of *Fasn*, *Cpt1α*, *Ppargc1α*, *Pnpla2* and *Lep* due to HF compared with NF feeding. *Lep* gene expression showed significant downregulation for HF + Q and HF + H mice compared with HF. No significant regulation by quercetin or hesperetin was observed for the other genes (Table 2).

**Table 2:** Gene expression in white adipose tissue

Gene symbol	Function	HF	HF + Q	HF + H
		versus NF	versus HF	
*Fasn*	Fatty acids synthesis	−4.19^###^	1.05	−1.18
*Cpt1α*	Fatty acid beta-oxidation	1.83^###^	−1.32	−1.22
*Ppargc1α*	mitochondrial biogenesis	−2.13^###^	1.10	−1.10
*Lipe*	Lipolysis	−1.10	1.02	−1.05
*Pnpla2*	Lipolysis	−1.66^###^	1.27	1.14
*Lep*	Leptin	5.80^###^	−1.70***	−1.52**
*Pparg*	Adipocyte differentiation	−1.48	−1.00	−1.14
*Clmp*	Adipocyte differentiation	1.25	−1.13	−1.20

Data are presented as mean fold change of HF versus NF and HF + flavonoids versus HF

NF, normal-fat diet; HF, high-fat diet; HF + Q, HF supplemented with quercetin; HF + H, hesperetin

^###^Indicates significant difference of HF to NF (*p* < 0.001), ** indicates a significant difference of HF + flavonoid to HF (*p* < 0.01), *** *p* < 0.001

### White adipose tissue and leptin

**Fig. 4:**
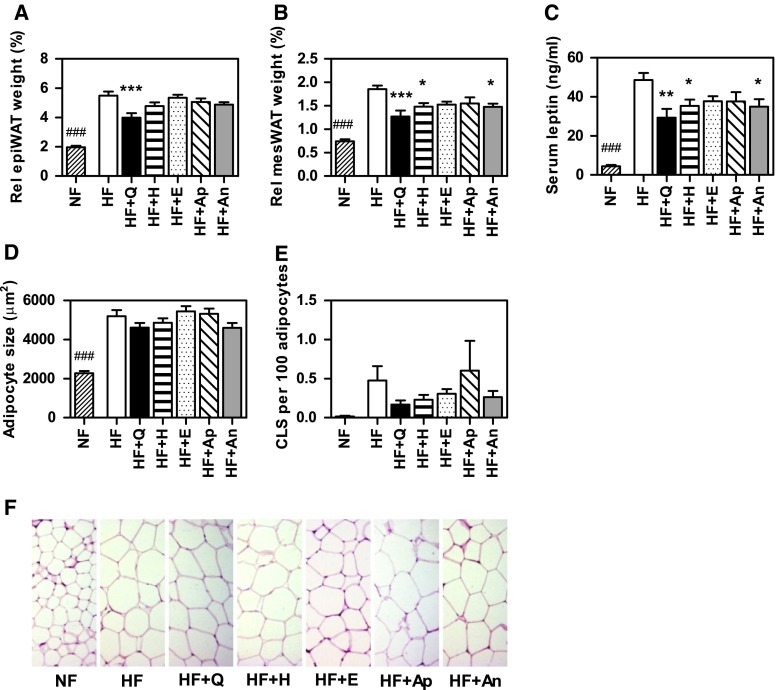
Effects of flavonoids on HF-induced effects on white adipose tissue weights, serum leptin levels, adipocyte size and crown-like structures in epiWAT. Mesenteric white adipose tissue (mesWAT, **a**) and epididymal tissue (epiWAT, **b**) relative weight (gram/gram body weight) at the end of the dietary intervention. Serum leptin levels (**c**). Quantification of adipocyte sizes (**d**) and crown-like structures (CLS) by macrophage staining (**e**) in epiWAT. Representative pictures of adipocyte stainings (**f**). Data are presented as mean ± SEM. *Asterisk* indicates a significant difference of HF + flavonoid to HF (*p* < 0.05); **p < 0.01; ***p < 0.001; *triple hash* indicates a significant difference of HF to NF (*p* < 0.001). NF, normal-fat diet; HF, high-fat diet; HF supplemented with quercetin, HF + Q; hesperetin, HF + H; epicatechin, HF + E; apigenin, HF + Ap; anthocyanins, HF + An

HF feeding significantly induced relative mesWAT and epiWAT weights compared with NF feeding. Supplementation with the flavonoids prevented this induction of relative mesWAT weight, which was significantly lower for HF + quercetin, HF + hesperetin and HF + anthocyanins compared with HF (Fig. 4a). Relative epiWAT was only significantly decreased for HF + quercetin versus HF (Fig. 4b). A trend for decreased relative WAT weights was seen for most of the HF + flavonoid diets compared with HF, in line with their reduced body weights.

Likewise, serum leptin levels, which are known to be correlated with total adipose tissue mass, showed significant higher values for HF mice compared with NF mice. Quercetin, hesperetin and anthocyanins supplementation to HF significantly lowered serum leptin levels compared with HF (Fig. 4c), which also corresponds to the gene expression results of *Lep*.

Adipocyte size (Fig. 4d) and macrophage infiltration (Fig. 4e) in epiWAT were determined, and representative pictures of adipocyte stainings are shown in Fig. 4f. Mean adipocyte size was significantly higher for HF mice versus NF mice. Remarkably, supplementation of flavonoids to the HF diet did not significantly affect the HF-induced increased adipocyte size, indicating that the lower observed WAT weights were not due to smaller adipocytes; consequently, the number of adipocytes should be decreased.

MAC-2 staining as a marker of macrophage infiltration of epiWAT revealed no indication for inflammation (Murano et al. 2008) for all groups.

### Correlations

**Fig. 5:**
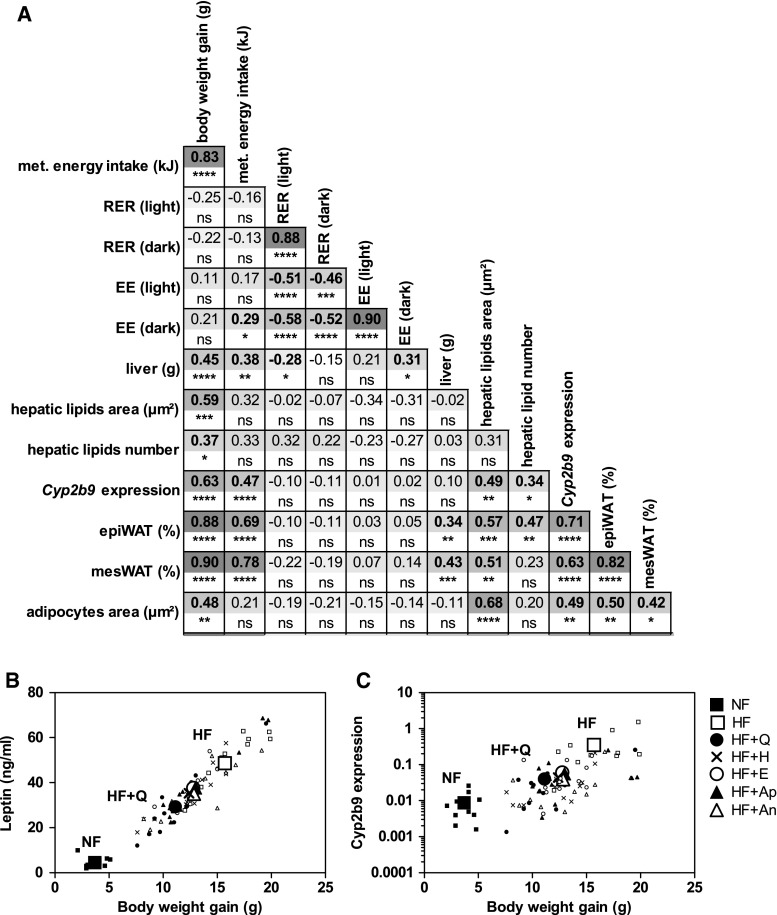
Correlations between measured parameters. Pearson correlations between body weight gain, metabolisable energy intake, RER, EE, liver weight, hepatic lipid accumulation, *Cyp2b9* expression, weight and area of WAT and serum leptin levels were analysed for HF and all HF + flavonoids groups (**a**). Pearson *r* values are presented, with underneath the significance of the correlation; *p < 0.05, **p < 0.01, ***p < 0.001, ****p < 0.0001. Correlation plot of BW gain to leptin for HF, HF + flavonoids and NF (**b**). Correlation plot of BW gain to *Cyp2b9* expression (log2) for HF, HF + flavonoids and NF (**c**). *Large symbols* indicate the mean of the value per group, and *small symbols* indicate the individual mice

Pearson correlations between all parameters measured showed significant correlations between body weight gain and all parameters for HF and all HF + flavonoid groups (Fig. 5a), except for the indirect calorimetry measurements (RER and EE). Strongest correlation was found between body weight gain and leptin (*r* = 0.94, *p* < 0.0001, Fig. 5b). Interestingly, *Cyp2b9* expression showed also a strong correlation (*p* < 0.001) with body weight gain (Fig. 5c), metabolisable energy intake, relative weights of epiWAT and mesWAT, and with serum leptin levels. The correlation plots shown in Fig. 5b, c indicate quercetin as most potent flavonoid to modify the HF-induced effects in the direction of NF effects.

## Discussion

As expected, HF (40 en% fat) induced adiposity, as seen by body weight gain, more visceral adipose tissue and hepatic lipid accumulation, compared with NF (10 en% fat). We examined whether this unfavourable change could be prevented by individual supplementation of HF with five different flavonoids: quercetin, hesperetin, epicatechin, apigenin or anthocyanins. All flavonoids reduced HF-induced body weight gain and hepatic *Cyp2b9* expression. Quercetin was the most potent flavonoid in beneficially affecting the HF-disturbed whole-body energy balance and lipid handling. In addition to quercetin, also hesperetin and anthocyanins decreased relative mesWAT weights and serum leptin levels. None of the flavonoids affected energy intake, substrate use and energy expenditure.

The reduced body weight gain by all flavonoids was not caused by reduced intake of metabolisable energy. As an example, for HF + quercetin-fed mice metabolisable intake was marginally lower than for HF mice, but this difference can only explain a lower body weight of ~0.5 g, based on the observed feed efficiency, instead of the observed reduction of 4.6 g after 12 weeks. Also activity and energy expenditure measurements in weeks 1, 5 and 11 of the intervention showed no differences between the HF + flavonoid groups and HF and could therefore not explain the differences in final body weight. These results correspond with previous data which showed that changes in energy expenditure could not explain weight differences due to quercetin supplementation in mice (Henagan et al. 2014; Stewart et al. 2008). Overall, energy intake, energy demand or energy loss were not different between the HF + flavonoid groups and the HF control group and therefore cannot explain the flavonoid-induced body weight differences.

The flavonoid-induced body weight differences could neither be explained by an increase in lipid oxidation, as suggested earlier (Hoek-van den Hil et al. 2013; Jung et al. 2013). Our indirect calorimetry measurements showed no indication for differences in substrate use between the different HF + flavonoid groups and HF. There was no lowering of the RER value, which would indicate increased fatty acid versus glucose oxidation. This confirms an earlier observation for quercetin supplementation showing an absence of effects on RER values (Stewart et al. 2008). Also serum lipid fractions were not significantly changed by the flavonoids; only some lipid fractions showed a small tendency to decrease into the direction of values observed for NF mice. Furthermore, except *Cyp2b9*, all studied lipid metabolism genes, in liver and WAT for any of the flavonoid supplementations showed no significant differences in expression levels compared with HF. This indicates that the flavonoids did not induce measurable changes in lipid metabolism, which therefore cannot serve as an explanation for the observed body weight differences.

Since individual parameters alone cannot explain the effect of the flavonoids on HF-induced increases in body weight, this effect may possibly be explained by several smaller changes that cumulatively contribute to the total body weight balance. Besides reduction in body weight gain, also lowering of serum leptin levels and relative mesenteric adipose tissue weights was found by supplementation of quercetin, epicatechin and anthocyanins. We therefore analysed to which extent the various parameters measured were correlated with each other. Body weight gain, WAT weights and leptin levels were strongly correlated (Fig. 5, *R* > 0.85, *p* < 0.001). Also, significant correlations were found for metabolisable energy intake and serum leptin levels. Furthermore, also on tissue level, WAT percentages, adipocytes area, hepatic lipid accumulation and hepatic *Cyp2b9* expression correlated with many of the measured parameters, including body weight gain. In contrast, energy expenditure or RER were not correlated with body weight gain. Overall, these correlation analyses confirm that energy expenditure and lipid oxidation alone cannot explain the body weight gain lowering effect of the flavonoids. These correlations imply that several effects on whole body level and tissue level occur, which are related to the body weight gain differences, and these effects could possibly cumulatively contribute to the weight lowering effects.

It is of interest to note that whole-body energy balance was studied throughout the experiment at several time points. Total energy intake, which was corrected for faecal losses, activity and energy expenditure were taken into account, providing a comprehensive picture of whole-body energy balance, a major strength of this study. In addition, liver as well as white adipose tissue were investigated, which gives an extensive overview in comparing the effects of the different flavonoids.

Additionally, the effects of flavonoid supplementations on the motor and gait performances was studied, because flavonoids are also suggested to have neuroprotective actions (Spencer 2009), which could lead to improved motor performance. No improvement was observed; however, also no indication for adverse effects on these performance parameters was obtained.

Furthermore, we measured the presence of the flavonoids in the diets and in serum, which showed absorption of quercetin and hesperetin. The other flavonoids were not detectable in serum, which could be ascribed to their pharmacokinetics known to include a relatively fast rate of elimination (Hollman 1997; Janssen et al. 1998; Manach et al. 2005).

Interestingly, hepatic *Cyp2b9* transcript levels were strongly downregulated by all flavonoids compared with HF (*p* < 0.01). *Cyp2b9* and *Cyp2b10* are the major mice homologues of human CYP2B6. Biochemical analysis has shown that CYP2B6 can be inhibited by anthocyanidins, one of the flavonoids used here (Srovnalova et al. 2014). These P450 enzymes are involved in metabolism of exogenous and endogenous compounds, such as steroid hormones, prostaglandins and fatty acids. Furthermore, *Cyp2b9* is known to be regulated by the transcription factor CAR (Honkakoski et al. 1998; Sueyoshi et al. 1999), which we previously proposed as a possible target of quercetin (Hoek-van den Hil et al. 2013, 2014). Importantly, HF increased *Cyp2b9* expression 40-fold compared with NF. This strong upregulation of *Cyp2b9* by HF suggests that the amount of lipids in the diet influenced the transcription level of *Cyp2b9*. In a HF dietary context, flavonoids downregulated *Cyp2b9* with fold changes ranging from 6 to 9. This decreased expression of *Cyp2b9* in the various HF + flavonoid groups as compared to the HF group positively correlated with reduced body weight gain, mesenteric WAT weight, serum leptin levels and hepatic lipid accumulation. These results suggest a relation of the effects of flavonoids on *Cyp2b9* with lipid homeostasis, which could be possibly regulated via CAR. Overall, the reduced *Cyp2b9* transcript levels by all flavonoids indicate a relation between *Cyp2b9* and the preventive effects of the flavonoids on the HF-induced effects. This suggests that *Cyp2b9* expression can be a marker in a possible common mode of action of the flavonoids. It is tempting to speculate that the newly observed common effects of the flavonoids on hepatic *Cyp2b9* expression and adiposity are mechanistically related, but this requires further investigation, as is the possible impact of the polymorphic CYP2B6 enzyme on CVD preventive lipid lowering effects of flavonoids.

Of all studied flavonoids, quercetin showed the strongest lowering effects on HF-induced parameters. Quercetin not only affected most parameters, but also showed the most prominent effects. This is supported by correlation analysis (Fig. 5a). It is particularly illustrated by the correlation plot of leptin versus body weight gain (Fig. 5b) and of *Cyp2b9* versus body weight gain (Fig. 5c), which indicates that quercetin better prevented the HF-induced effects resulting in values closer to those observed for mice fed NF.

Furthermore, the strong correlation of leptin and body weight gain for all HF + flavonoid groups suggests that leptin can be used as a sensitive marker for the effects on adiposity. This is especially of interest given that leptin, known to regulate body weight gain, can be measured easily and rapidly in circulation. This positions leptin as a potential practical and useful marker to quantify flavonoid effects on HF-induced adiposity, to be used in animal as well as human studies.

The doses of the individual flavonoids provided ~320–500 mg/kg bw/day in this study. In humans, the estimated intake of these individual flavonoids via food is between 0.02 (for apigenin) and 3 mg/kg bw/day (for anthocyanins), and supplementary intake up to 23 mg/kg bw/day (Manach et al. 2004). The levels used in this experiment are comparable with levels used in animal studies; however, for further research it would be interesting to elaborate if lower doses can also be effective.

We have investigated the effects of individual flavonoids. However, in our daily diet a mixture of flavonoids will be present. In theory flavonoids could act in an additive or synergistic way, the latter for example due to their influence on efflux transporters. Furthermore, the dietary context could also influence the effects of flavonoids (Bohn 2014; Hoek-van den Hil et al. 2014).

In conclusion, a direct comparison of metabolic effects of quercetin, hesperetin, epicatechin, apigenin and anthocyanins indicated that all flavonoids beneficially affected HF-induced disturbance of whole-body energy balance and lipid handling, with serum leptin levels as a sensitive marker. This confirms the suggested potential of these flavonoids in lowering CVD risk factors. Furthermore, the reduction in hepatic *Cyp2b9* transcript levels was shown for all flavonoids. Overall, quercetin appeared to be the most potent flavonoid in preventing HF-induced effects.

## Electronic supplementary material

Supplementary material 1 (PDF 549 kb)

## References

[CR1] Benavente-Garcia O, Castillo J (2008). Update on uses and properties of citrus flavonoids: new findings in anticancer, cardiovascular, and anti-inflammatory activity. J Agric Food Chem.

[CR2] Boden G (2008). Obesity and free fatty acids. Endocrinol Metab Clin North Am.

[CR3] Bohn T (2014). Dietary factors affecting polyphenol bioavailability. Nutr Rev.

[CR4] Chong MF, Macdonald R, Lovegrove JA (2010). Fruit polyphenols and CVD risk: a review of human intervention studies. Br J Nutr.

[CR5] de Boer VC, van Schothorst EM, Dihal AA, van der Woude H, Arts IC, Rietjens IM, Hollman PC, Keijer J (2006). Chronic quercetin exposure affects fatty acid catabolism in rat lung. Cell Mol Life Sci.

[CR7] Estruch R, Ros E, Salas-Salvado J, Covas MI, Corella D, Aros F, Gomez-Gracia E, Ruiz-Gutierrez V, Fiol M, Lapetra J, Lamuela-Raventos RM, Serra-Majem L, Pinto X, Basora J, Munoz MA, Sorli JV, Martinez JA, Martinez-Gonzalez MA, Investigators PS (2013). Primary prevention of cardiovascular disease with a Mediterranean diet. N Engl J Med.

[CR8] Harchaoui KE, Visser ME, Kastelein JJ, Stroes ES, Dallinga-Thie GM (2009). Triglycerides and cardiovascular risk. Curr Cardiol Rev.

[CR9] Henagan TM, Lenard NR, Gettys TW, Stewart LK (2014). Dietary quercetin supplementation in mice increases skeletal muscle PGC1alpha expression, improves mitochondrial function and attenuates insulin resistance in a time-specific manner. PLoS One.

[CR6] den Hoek-van den Hil EF, Beekmann K, Keijer J, Hollman PCH, Rietjens IMCM, van Schothorst EM (2012). Interference of flavonoids with enzymatic assays for the determination of free fatty acid and triglyceride levels. Anal Bioanal Chem.

[CR10] Hoek-van den Hil EF, Keijer J, Bunschoten A, Vervoort JJ, Stankova B, Bekkenkamp M, Herreman L, Venema D, Hollman PC, Tvrzicka E, Rietjens IM, van Schothorst EM (2013). Quercetin induces hepatic lipid omega-oxidation and lowers serum lipid levels in mice. PLoS One.

[CR11] Hoek-van den Hil EF, van Schothorst EM, van der Stelt I, Swarts HJM, Venema D, Sailer M, Vervoort JJM, Hollman PCH, Rietjens IMCM, Keijer J (2014). Quercetin decreases high-fat diet induced body weight gain and accumulation of hepatic and circulating lipids in mice. Genes Nutr.

[CR12] Hoevenaars FP, van Schothorst EM, Horakova O, Voigt A, Rossmeisl M, Pico C, Caimari A, Kopecky J, Klaus S, Keijer J (2012). BIOCLAIMS standard diet (BIOsd): a reference diet for nutritional physiology. Genes Nutr.

[CR13] Hoevenaars FP, Keijer J, Swarts HJ, Snaas-Alders S, Bekkenkamp-Grovenstein M, van Schothorst EM (2013). Effects of dietary history on energy metabolism and physiological parameters in C57BL/6J mice. Exp Physiol.

[CR14] Hoevenaars FP, Keijer J, Herreman L, Palm I, Hegeman MA, Swarts HJ, van Schothorst EM (2014). Adipose tissue metabolism and inflammation are differently affected by weight loss in obese mice due to either a high-fat diet restriction or change to a low-fat diet. Genes Nutr.

[CR15] Hollman PC (1997). Bioavailability of flavonoids. Eur J Clin Nutr.

[CR16] Honkakoski P, Zelko I, Sueyoshi T, Negishi M (1998). The nuclear orphan receptor CAR-retinoid X receptor heterodimer activates the phenobarbital-responsive enhancer module of the CYP2B gene. Mol Cell Biol.

[CR17] Hooper L, Kroon PA, Rimm EB, Cohn JS, Harvey I, Le Cornu KA, Ryder JJ, Hall WL, Cassidy A (2008). Flavonoids, flavonoid-rich foods, and cardiovascular risk: a meta-analysis of randomized controlled trials. Am J Clin Nutr.

[CR18] Janssen K, Mensink RP, Cox FJ, Harryvan JL, Hovenier R, Hollman PC, Katan MB (1998). Effects of the flavonoids quercetin and apigenin on hemostasis in healthy volunteers: results from an in vitro and a dietary supplement study. Am J Clin Nutr.

[CR19] Jung CH, Cho I, Ahn J, Jeon TI, Ha TY (2013). Quercetin reduces high-fat diet-induced fat accumulation in the liver by regulating lipid metabolism genes. Phytother Res PTR.

[CR20] Kim A, Chiu A, Barone MK, Avino D, Wang F, Coleman CI, Phung OJ (2011). Green tea catechins decrease total and low-density lipoprotein cholesterol: a systematic review and meta-analysis. J Am Diet Assoc.

[CR21] Manach C, Scalbert A, Morand C, Remesy C, Jimenez L (2004). Polyphenols: food sources and bioavailability. Am J Clin Nutr.

[CR22] Manach C, Williamson G, Morand C, Scalbert A, Remesy C (2005). Bioavailability and bioefficacy of polyphenols in humans. I. Review of 97 bioavailability studies. Am J Clin Nutr.

[CR23] Massaro M, Scoditti E, Carluccio MA, De Caterina R (2010). Nutraceuticals and prevention of atherosclerosis: focus on omega-3 polyunsaturated fatty acids and mediterranean diet polyphenols. Cardiovasc Ther.

[CR24] Mclean JA, Tobin G (1987). Animal and human calorimetry.

[CR25] Murano I, Barbatelli G, Parisani V, Latini C, Muzzonigro G, Castellucci M, Cinti S (2008). Dead adipocytes, detected as crown-like structures, are prevalent in visceral fat depots of genetically obese mice. J Lipid Res.

[CR26] Perez-Vizcaino F, Duarte J (2010). Flavonols and cardiovascular disease. Mol Aspects Med.

[CR27] Peronnet F, Massicotte D (1991). Table of nonprotein respiratory quotient—an update. Can J Sport Sci.

[CR28] Peterson JJ, Dwyer JT, Jacques PF, McCullough ML (2012). Associations between flavonoids and cardiovascular disease incidence or mortality in European and US populations. Nutr Rev.

[CR29] Phung OJ, Baker WL, Matthews LJ, Lanosa M, Thorne A, Coleman CI (2010). Effect of green tea catechins with or without caffeine on anthropometric measures: a systematic review and meta-analysis. Am J Clin Nutr.

[CR030] Qin Y, Xia M, Ma J, Hao Y, Liu J, Mou H, Cao L, Ling W (2009). Anthocyanin supplementation improves serum LDL- and HDL-cholesterol concentrations associated with the inhibition of cholesteryl ester transfer protein in dyslipidemic subjects. Am J Clin Nutr.

[CR30] Scorletti E, Calder PC, Byrne CD (2011). Non-alcoholic fatty liver disease and cardiovascular risk: metabolic aspects and novel treatments. Endocrine.

[CR31] Slob W (2002). Dose-response modeling of continuous endpoints. Toxicol Sci.

[CR32] Spencer JP (2009). Flavonoids and brain health: multiple effects underpinned by common mechanisms. Genes Nutr.

[CR33] Srovnalova A, Svecarova M, Zapletalova MK, Anzenbacher P, Bachleda P, Anzenbacherova E, Dvorak Z (2014). Effects of anthocyanidins and anthocyanins on the expression and catalytic activities of CYP2A6, CYP2B6, CYP2C9, and CYP3A4 in primary human hepatocytes and human liver microsomes. J Agric Food Chem.

[CR34] Stewart LK, Soileau JL, Ribnicky D, Wang ZQ, Raskin I, Poulev A, Majewski M, Cefalu WT, Gettys TW (2008). Quercetin transiently increases energy expenditure but persistently decreases circulating markers of inflammation in C57BL/6J mice fed a high-fat diet. Metabolism.

[CR35] Sueyoshi T, Kawamoto T, Zelko I, Honkakoski P, Negishi M (1999). The repressed nuclear receptor CAR responds to phenobarbital in activating the human CYP2B6 gene. J Biol Chem.

