# Supplementary material for: Direct comparison of metabolic health effects of the flavonoids quercetin, hesperetin, epicatechin, apigenin and anthocyanins in high-fat-diet-fed mice
Source: Genes Nutr. 2015 May 29;10(4):23. doi: 10.1007/s12263-015-0469-z (PMC4447677; doi:10.1007/s12263-015-0469-z)
Supplement: Supplementary file 1 — Supplementary material 1 (PDF 549 kb) [file 12263_2015_469_MOESM1_ESM.pdf]

Supplementary Information

Genes & Nutrition

Direct comparison of metabolic health effects of the flavonoids quercetin, hesperetin, epicatechin, apigenin and anthocyanins in high-fat-diet-fed mice

Elise F. Hoek-van den Hil[1,2,3], Evert M. van Schothorst*[1], Inge van der Stelt[1], Hans J.M. Swarts[1], Marjanne van Vliet[1], Tom Amolo[1], Jacques J.M. Vervoort[4], Dini Venema[3], Peter C.H. Hollman[3], Ivonne M.C.M. Rietjens[2], and Jaap Keijer[1].

[1] Human and Animal Physiology, Wageningen University, Wageningen, The Netherlands

[2] Division of Toxicology, Wageningen University, Wageningen, The Netherlands

[3] RIKILT Wageningen UR, Wageningen, The Netherlands

[4] Laboratory of Biochemistry, Wageningen University, Wageningen, The Netherlands

*Corresponding author: Evert M. van Schothorst, PhD,

Human and Animal Physiology, Wageningen University,

De Elst 1, 6708 WD Wageningen, The Netherlands

Tel.: +31-317-484699 Fax: + 31-317 483962

E-mail: evert.vanschothorst@wur.nl

Supplementary Table S1

Table S1. Sequences of the primers used for RT-qPCR

Gene Symbol	Forward primer (5'-->3')	Reverse primer (5'-->3')	Anealing temp (°C)
Acot3	GCTGTGACCTACCTGCTCAGTCA	ATATAGAGCCATTGATGATGACAGCGG	60
B2m	CCCCACTGAGACTGATACATACGC	AGAAACTGGATTTGTAATTAAGCAGGTTC	62
Clmp	CAGTGTGAGTCTGCCTCTG	CAGTCACCCGTACCACAC	58
Cpt1a	AAAGATCAATCGGACCCTAGACA	CAGCGAGTAGCGCATAGTCA	57
Csad	CCATGTGGATGCTGCCTGGGG	AAGAGCGGAGCACTGCAGCC	56.7
Cyp2b9	ACCACAAGCGCCACCCTCCA	TGGTAGCCGGTGTGAGCCGAT	55.7
Cyp4a10	TCTACCCACCTGTCCCAGGC	ACACCTCTGGATTTGGCCACA	58
Cyp4a14	TTCTTTCGCCTGCGGAATGC	CACTCCATCTGTGTGCTCGTGA	62
Fabp5	GTGGCGCCTGATGGAAAGCC	TCCACGATCATCTTCCCATCCTTCA	58
Fasn	AGTTAGAGCAGGACAAGCCCAAG	GTGCAGAGCTGTGCTCCTGA	55
Hao2	TGTTGAAGGCACTAGCCCTTGGAG	AGGTCTGGACTGATCTCAGCAACTG	58
Hprt1	TGACACTGGTAAAACAATGCAAACTTTG	GAGGTCCTTTTCACCAGCAAGCT	62
Lep	GGCTTTGGTCCTATCTGTCTTATGTTC	CCCTCTGCTTGGCGGATACC	60
Lipe	TGGAACTAAGTGGACGCAAGCC	TCAAGGTATCTGTGCCCAGTAAGC	58
Pnpla2	ACCACCCTTTCCAACATGCTACC	GCTACCCGTCTGCTCTTTCATCC	58
Pparg	ACCACTCGCATTCCTTTGAC	AAGGCACTTCTGAAACCGAC	63.5
Ppargc1a	CCCTGCCATTGTTAAGACC	TGCTGCTGTTCCTGTTTTC	60
Por	CGAGGGCAAGGAGCTGTACC	CACAGGTGGTCGATGGGTGG	62
Tfrc	CCTTGCACTCTTTGGACATGCTCATC	AACCCTGATGACTGAGATGGCGG	60

Primers were designed using the NCBI Primer-Blast (NCBI Web site) or based on literature for *Ppargc1a*[1] , *Clmp*[2]. *Acot3*, acyl-CoA thioesterase 3; *B2m*, Beta-2 microglobulin; *Clmp*, CXADR-like membrane protein; *Cpt1a*, Carnitine palmitoyltransferase 1A; *Csad*, Cysteine sulfinic acid decarboxylase; *Cyp2b9*, Cytochrome P450 family 2b9; *Cyp4a10*, Cytochrome P450 family 4a10; *Cyp4a14*, cytochrome P450 family 4a14; *Fabp5*, Fatty acid binding protein 5 epidermal; *Fasn*, Fatty acid synthase; *Hao2*, Hydroxyacid oxidase 2; *Hprt1*, hypoxanthine phophoribosyltransferase 1; *Lep*, Leptin; *Lipe*, Lipase, hormone sensitive; *Pnpla2*, Patatin-like phospholipase domain; *Pparg*, Peroxisome proliferator activated receptor gamma; *Ppargc1a*, Peroxisome proliferator activated receptor; *Por*, P450 (cytochrome) oxidoreductase; *Tfrc*, transferrin receptor.

[1] Wankhade UD, Vella KR, Fox DL, Good DJ (2010) Deletion of Nhlh2 results in a defective torpor response and reduced Beta adrenergic receptor expression in adipose tissue. PLoS One 5: e12324.
[2] Sze KL, Lui WY, Lee WM (2008) Post-transcriptional regulation of CLMP mRNA is controlled by tristetraprolin in response to TNFalpha via c-Jun N-terminal kinase signalling. Biochem J 410: 575-583.

Supplementary Table S2

Table S2. Flavonoid quantification in diets at the start of the experiment stored at -20 °C and after one week at room temperature (RT).

	Theoretical amount of flavonoid in the diet	Flavonoid start levels		Flavonoid levels after one week at RT	
	(%)	(ug/ml)	(% of theoretical amount)	(ug/ml)	(% of theoretical amount)
Quercetin	0,33	14.6	88	15.1	92
Hesperetin	0,33	31.8	97	30.7	93
Epicatechin	0,32	31.2	99	31.6	100
Apigenin	0,29	13.6	92	14.3	95
Anthocyanins	0,5	72.3	87	75.0	90

Data are presented as measured (0.5 g diet dissolved in 100 ml in case of quercetin and apigenin, in 50 ml in case of hesperetin and epicatechin, and in 30 ml in case of anthocyanins) and in percentage of the theoretical amount of flavonoids added to the diets.

Supplementary Table S3

Table S3. Gait analysis with catwalk in week 10.

Parameter	NF	HF	HF+Q	HF+H	HF+E	HF+Ap	HF+An
Run Duration (s)	2.89	3.35	3.28	3.09	3.21	3.09	3.06
RF Stand (s)	0.17	0.20	0.19	0.19	0.19	0.19	0.19
RF StandIndex	-4.79	-3.84	-3.88	-4.08	-3.97	-4.15	-4.12
RF MaxContactAt (%)	35.80	35.83	34.95	36.73	35.16	35.78	37.83
RF MaxContactArea (cm²)	0.22	0.25	0.24	0.25	0.25	0.24	0.24
RF MaxContactMaxIntensity	139.78	145.70	142.61	145.27	143.50	143.00	147.11
RF MaxContactIntensity	71.09	72.95	71.45	72.41	72.81	71.69	72.94
RF PrintLength (cm)	0.84	0.87	0.84	0.84	0.86	0.84	0.86
RF PrintWidth (cm)	0.75	0.78	0.76	0.76	0.79	0.76	0.75
RF PrintArea (cm²)	0.30	0.33	0.32	0.32	0.32	0.31	0.32
RF MaxIntensityAt (%)	55.19	61.04	57.58	61.06	59.83	60.23	61.34
RF MaxIntensity	148.73	157.95	153.53	157.14	155.60	155.13	159.49
RF MinIntensity	36.52	36.34	36.49	36.38	36.48	36.37	36.44
RF Intensity	74.70	77.34	75.61	76.92	77.01	75.86	76.82
RF IntensityOfThe15MostIntensePixels	114.00	120.52	116.98	119.92	119.54	116.59	120.82
RF Swing (s)	0.13	0.14	0.14	0.14	0.14	0.14	0.14
RF SwingSpeed (cm/s)	53.67	51.58	49.04	48.65	49.74	48.66	47.79
RF StrideLength (cm)	6.88	6.79	6.69	6.59	6.72	6.40	6.55
RF StepCycle (s)	0.30	0.33	0.34	0.33	0.33	0.32	0.33
RF DutyCycle (%)	55.15	58.46	56.65	56.64	56.97	57.45	56.50
RF SingleStance (s)	0.13	0.14	0.14	0.14	0.14	0.14	0.14
RF InitialDualStance (s)	0.02	0.03	0.03	0.03	0.03	0.03	0.02
RF TerminalDualStance (s)	0.02	0.03	0.02	0.03	0.02	0.03	0.03
RH Stand (s)	0.14[###]	0.18	0.17	0.17	0.17	0.17	0.17
RH StandIndex	-8.72[###]	-6.37	-6.72	-6.78	-6.60	-7.02	-6.62
RH MaxContactAt (%)	28.28	27.02	26.75	25.78	27.12	28.10	27.40
RH MaxContactArea (cm²)	0.20[#]	0.24	0.25	0.25	0.25	0.24	0.25
RH MaxContactMaxIntensity	162.92	172.67	173.94	170.76	169.42	171.17	168.14
RH MaxContactIntensity	79.27	82.09	82.87	82.49	80.98	80.97	81.57
RH PrintLength (cm)	0.79[#]	0.86	0.88	0.83	0.87	0.86	0.86
RH PrintWidth (cm)	0.68	0.75	0.74	0.72	0.76	0.75	0.76
RH PrintArea (cm²)	0.26[##]	0.32	0.33	0.31	0.32	0.31	0.32
RH MaxIntensityAt (%)	45.71	52.42	52.44	48.88	52.16	52.45	52.23
RH MaxIntensity	172.38[#]	182.99	184.92	179.76	179.63	180.92	178.84
RH MinIntensity	39.45	38.31	38.82	39.22	39.35	38.40	38.99
RH Intensity	83.99	87.22	87.60	87.73	86.24	85.92	86.83
RH IntensityOfThe15MostIntensePixels	132.83	146.12	147.84	144.97	144.10	143.35	144.04
RH Swing (s)	0.13	0.14	0.14	0.13	0.13	0.14	0.13
RH SwingSpeed (cm/s)	46.73	46.56	44.61	44.30	46.58	43.98	44.80
RH StrideLength (cm)	6.10	6.24	6.00	5.89	5.91	5.94	5.95
RH StepCycle (s)	0.27	0.31	0.30	0.30	0.29	0.30	0.30
RH DutyCycle (%)	49.77[##]	54.71	53.73	53.87	54.90	53.21	53.80
RH SingleStance (s)	0.12	0.13	0.13	0.13	0.12	0.12	0.12
RH InitialDualStance (s)	0.01	0.02	0.02	0.02	0.02	0.02	0.02
RH TerminalDualStance (s)	0.01	0.03	0.03	0.03	0.02	0.02	0.03
LF Stand (s)	0.17	0.19	0.19	0.19	0.19	0.19	0.19
LF StandIndex	-4.48	-4.16	-3.89	-3.94	-3.87	-4.02	-3.82
LF MaxContactAt (%)	34.41	34.55	34.73	35.89	35.32	34.45	34.01
LF MaxContactArea (cm²)	0.22[##]	0.26	0.24	0.25	0.24	0.24	0.24
LF MaxContactMaxIntensity	136.84	145.75	142.53	146.92	145.99	143.82	144.91
LF MaxContactIntensity	70.40	72.39	71.61	72.87	72.52	71.50	72.00
LF PrintLength (cm)	0.82	0.86	0.83	0.86	0.84	0.84	0.84
LF PrintWidth (cm)	0.74	0.76	0.76	0.76	0.75	0.76	0.79
LF PrintArea (cm²)	0.30	0.33	0.31	0.33	0.31	0.31	0.31
LF MaxIntensityAt (%)	57.58	61.34	59.85	61.03	59.17	60.41	60.56
LF MaxIntensity	148.27	158.10	155.26	158.92	157.39	156.20	156.83
LF MinIntensity	36.69	36.28	36.36	36.55	36.36	36.37	36.40
LF Intensity	74.54	76.73	75.80	77.22	76.66	75.82	75.88
LF IntensityOfThe15MostIntensePixels	113.01	120.18	117.42	120.85	119.28	117.27	118.14
LF Swing (s)	0.14	0.14	0.14	0.14	0.14	0.14	0.14
LF SwingSpeed (cm/s)	52.78	49.44	48.25	49.67	49.72	47.88	48.89
LF StrideLength (cm)	6.84	6.80	6.63	6.54	6.71	6.44	6.55
LF StepCycle (s)	0.30	0.34	0.33	0.33	0.33	0.33	0.33
LF DutyCycle (%)	54.48	56.90	56.21	57.46	57.02	56.51	57.30
LF SingleStance (s)	0.13	0.14	0.14	0.14	0.14	0.14	0.14

Continuing of Supplementary Table S3

Parameter	NF	HF	HF+Q	HF+H	HF+E	HF+Ap	HF+An
LF InitialDualStance (s)	0.02	0.03	0.02	0.03	0.02	0.03	0.03
LF TerminalDualStance (s)	0.02	0.03	0.02	0.03	0.03	0.03	0.02
LH Stand (s)	0.14$^{\#\#}$	0.17	0.16	0.17	0.17	0.17	0.17
LH StandIndex	-9.49$^{\#\#}$	-7.13	-6.69	-7.43	-6.65	-6.67	-6.48
LH MaxContactAt (%)	29.10	28.82	27.01	27.45	26.04	26.46	26.87
LH MaxContactArea (cm^2)	0.21$^{\#\#}$	0.24	0.25	0.27	0.26	0.24	0.23
LH MaxContactMaxIntensity	167.66	174.38	171.56	178.64	174.68	171.47	165.12
LH MaxContactIntensity	82.46	82.72	82.00	84.02	82.49	82.22	79.47
LH PrintLength (cm)	0.81	0.84	0.86	0.87	0.87	0.83	0.85
LH PrintWidth (cm)	0.67	0.74	0.76	0.76	0.77	0.74	0.75
LH PrintArea (cm^2)	0.26$^{\#}$	0.31	0.32	0.34	0.33	0.31	0.31
LH MaxIntensityAt (%)	47.27	51.38	52.68	48.95	53.20	49.70	51.83
LH MaxIntensity	176.53	183.79	181.55	186.84	184.83	180.92	176.39
LH MinIntensity	39.74	38.16	38.25	38.72	38.48	39.37	38.63
LH Intensity	87.05	87.91	86.84	89.33	87.40	87.30	85.20
LH IntensityOfThe15MostIntensePixels	137.75	147.96	145.69	151.88	148.60	144.29	140.91
LH Swing (s)	0.14	0.14	0.13	0.14	0.14	0.13	0.13
LH SwingSpeed (cm/s)	44.07	45.62	44.41	44.62	44.90	44.18	45.78
LH StrideLength (cm)	6.13	6.17	5.93	6.04	6.12	5.68	5.82
LH StepCycle (s)	0.27	0.30	0.30	0.31	0.30	0.29	0.29
LH DutyCycle (%)	48.48$^{\#\#}$	53.62	53.46	53.39	53.26	54.13	54.77
LH SingleStance (s)	0.12	0.12	0.12	0.13	0.12	0.12	0.12
LH InitialDualStance (s)	0.01	0.03	0.02	0.02	0.02	0.02	0.02
LH TerminalDualStance (s)	0.01	0.02	0.02	0.02	0.03	0.03	0.02
StepSequence NumberOfPatterns	29.42	30.92	30.83	31.83	30.83	32.08	31.75
StepSequence CA (%)	23.15	18.25	22.07	27.74	17.81	24.65	27.37
StepSequence CB (%)	19.25	25.14	28.57	19.55	26.36	22.58	18.02
StepSequence AA (%)	10.19	9.05	9.85	11.63	11.74	9.85	10.80
StepSequence AB (%)	42.88	46.23	37.14	38.97	39.71	37.94	40.98
StepSequence RA (%)	1.87	1.04	1.08	1.06	1.35	3.19	2.04
StepSequence RB (%)	2.65$^{\#}$	0.28	1.29	1.05	3.05*	1.79	0.78
StepSequence RegularityIndex (%)	92.64	92.92	92.30	92.33	93.47	92.12	93.12
BOS FrontPaws (cm)	1.20	1.21	1.20	1.22	1.27	1.20	1.26
BOS HindPaws (cm)	2.66	2.96	2.76	2.78	2.85	2.87	2.85
Average Speed	22.49	20.03	19.91	20.06	19.87	19.33	19.65
Maximum Variation (%)	20.89	21.46	23.27	21.19	22.21	23.74	21.25
Cadence	13.65	12.21	12.33	12.37	12.41	12.58	12.46
NumberOfSteps	148.00	152.00	152.58	158.75	151.25	159.08	156.17
PrintPositions RightPaws (cm)	1.23	1.12	1.25	1.23	1.29	1.13	1.33
PrintPositions LeftPaws (cm)	1.12	1.27	1.21	1.16	1.14	1.53	1.46
PhaseDispersions RF->LH	16.41	24.67	8.35	14.74	8.71	10.40	15.65
PhaseDispersions LF->RH	9.45	24.40	16.64	14.98	15.33	7.66	8.77
PhaseDispersions LH->RH	38.70	39.85	38.88	38.11	37.97	38.85	38.08
PhaseDispersions LF->RF	50.03	49.02	50.01	50.56	49.12	49.44	51.21
PhaseDispersions RF->RH	47.29	50.66	47.73	48.17	47.48	49.22	48.01
PhaseDispersions LF->LH	46.96	49.44	47.42	48.99	49.75	45.95	47.57
Couplings RF->LH	21.35	28.60	13.79	20.54	12.91	16.22	21.53
Couplings LF->RH	14.31	29.96	22.47	19.69	22.48	13.57	13.31
Couplings LH->RF	17.81	26.75	24.39	25.42	31.06	15.32	16.92
Couplings RH->LF	25.87	27.48	25.00	24.20	25.03	17.47	8.38
Couplings LH->RH	45.65	45.28	44.69	46.24	43.22	45.07	45.02
Couplings LF->RF	49.57	48.64	49.85	50.75	48.89	48.83	50.78
Couplings RH->LH	44.48	45.79	44.79	45.57	46.97	43.38	43.69
Couplings RF->LH	50.08	51.17	49.80	49.11	50.84	50.35	49.00
Couplings RF->RH	49.59	50.93	48.84	49.86	48.66	50.44	49.52
Couplings LF->LH	49.87	50.14	49.20	50.51	50.70	46.71	48.43
Couplings RH->RF	44.51	45.74	47.83	46.55	47.07	45.18	45.87
Couplings LH->LF	45.63	47.10	46.74	45.81	46.26	47.65	46.82
Support Zero (%)	0.15	0.06	0.05	0.02	0.03	0.03	0.02
Support Single (%)	5.90$^{\#\#\#}$	2.02	2.94	1.90	2.64	2.78	2.21
Support Diagonal (%)	73.54	69.45	71.44	70.70	70.24	69.06	71.57
Support Girdle (%)	3.44$^{\#}$	2.08	2.47	2.23	1.43	2.67	1.65
Support Lateral (%)	2.09	1.19	1.14	1.33	1.48	1.61	1.02
Support Three (%)	13.48$^{\#\#}$	21.97	19.10	20.25	20.53	19.90	20.02
Support Four (%)	1.39	3.23	2.87	3.56	3.65	3.94	3.52

Parameters were not significantly different between HF+flavonoids groups and HF. Some parameters were significantly different between HF and NF, which are indicates with $^{\#}$($p<0.05$), $^{\#\#}$($p<0.01$), $^{\#\#\#}$($p<0.001$). RF; right front paw, RH; right hind paw, LF; left front paw, LH; left hind paw, BOS; base of support.

Supplementary Table S4

Table S4. 24-hour indirect calorimetry measurements of RER and EE in week 11.

**Indirect calorimetry**		**NF**	**HF**	**HF+Q**	**HF+H**	**HF+E**	**HF+Ap**	**HF+An**
**RER**	dark	1.00# ± 0.07	0.87 ± 0.05	0.84 ± 0.07	0.87 ± 0.07	0.86 ± 0.05	0.87 ± 0.07	0.87 ± 0.05
	light	0.94# ± 0.08	0.83 ± 0.07	0.82 ± 0.08	0.85 ± 0.06	0.83 ± 0.06	0.85 ± 0.06	0.83 ± 0.07
**EE**	dark	0.53 ± 0.06	0.55 ± 0.06	0.54 ± 0.06	0.56 ± 0.06	0.54 ± 0.07	0.56 ± 0.06	0.55 ± 0.06
**(kcal/h)**	light	0.44 ± 0.06	0.48 ± 0.06	0.48 ± 0.05	0.48 ± 0.06	0.47 ± 0.07	0.48 ± 0.06	0.48 ± 0.06

Indirect calorimetric data are shown for both 12-hour dark and 12-hour light phase. Data are presented as mean ± SD (n=9). # indicates significant difference of HF to NF (p<0.05). RER; respiratory exchange ratio, EE; Energy Expenditure, NF; normal-fat diet, HF; high-fat diet, HF+Q; HF supplemented with quercetin, HF+H; HF supplemented with hesperetin, HF+E; HF supplemented with epicatechin, HF+Ap; HF supplemented with apigenin, HF+An; HF supplemented with anthocyanins.

Supplementary Figure S1

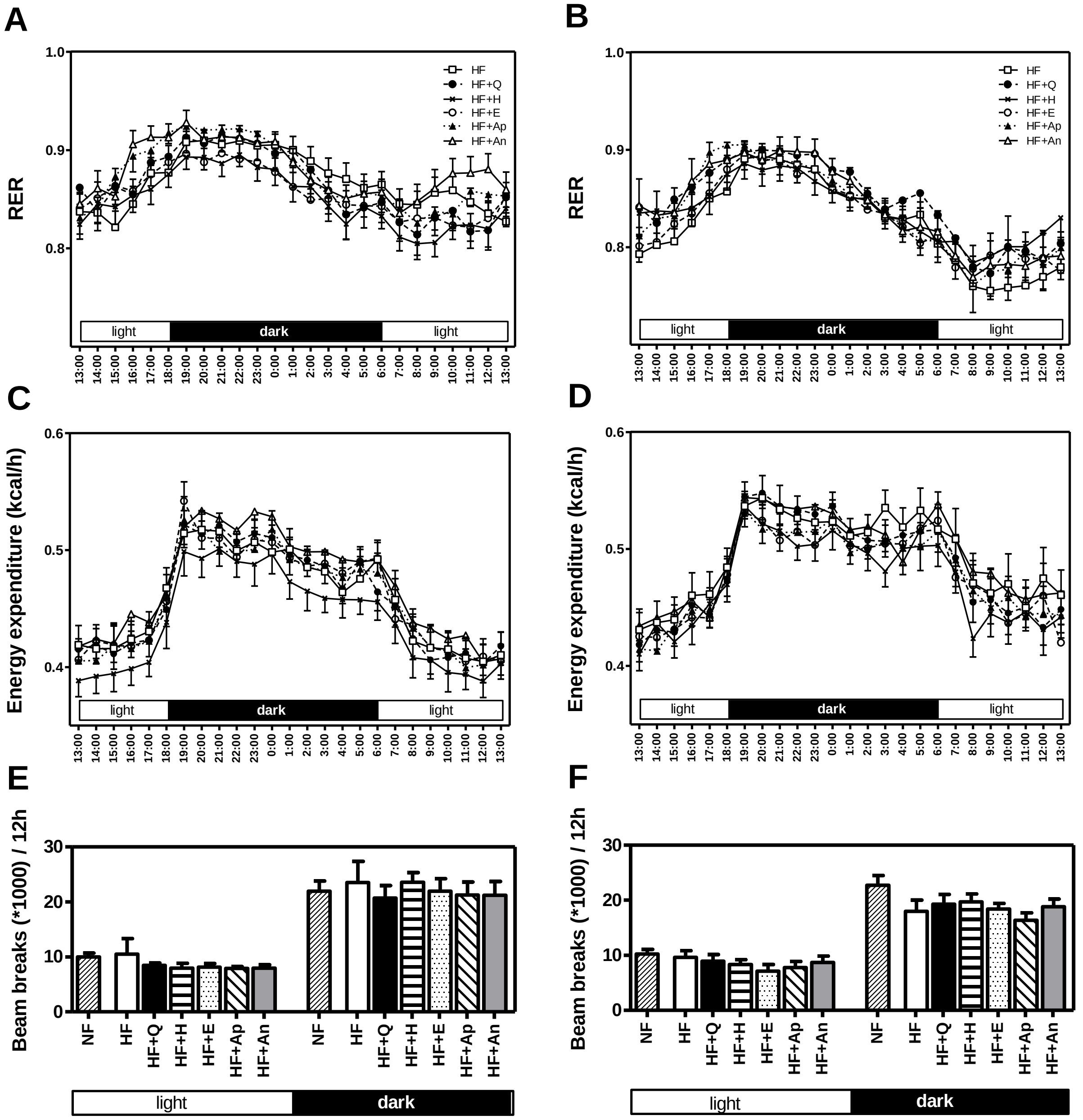

Figure S1. Whole body RER, energy expenditure, and activity levels were not affected by flavonoid supplementation in week 1 and 5. Indirect calorimetry measurements of 24 hour RER in week 1 (A) and in week 5 (B) and energy expenditure in week 1 (C) and in week 5 (D). Activity measured during indirect calorimetry measurements for light (12 h) and dark (12h) period in week 1 (E) and in week 5 (f). Data are presented as mean ± SEM. # indicates a significant difference of NF to HF. NF, normal fat diet; HF, high-fat diet; HF+Q, HF supplemented with quercetin; HF+H, HF supplemented with hesperetin; HF+E, HF supplemented with epicatechin; HF+Ap, HF supplemented with apigenin; HF+An, HF supplemented with anthocyanins; RER, respiration exchange ratio.

Supplementary Figure S2

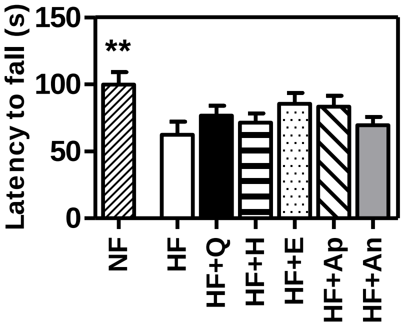

Figure S2. Rotarod measurements in week 9.
Data is presented as latency to fall in seconds
